# *In silico *investigation of ADAM12 effect on TGF-*β *receptors trafficking

**DOI:** 10.1186/1756-0500-2-193

**Published:** 2009-09-24

**Authors:** Jérémy Gruel, Michel LeBorgne, Nolwenn LeMeur, Nathalie Théret

**Affiliations:** 1 EA 4427 SeRAIC/INSERM U620, Université de Rennes 1, Rennes, France; 2 IRISA - Symbiose, Université de Rennes 1, Rennes, France

## Abstract

**Background:**

The transforming growth factor beta is known to have pleiotropic effects, including differentiation, proliferation and apoptosis. However the underlying mechanisms remain poorly understood. The regulation and effect of TGF-*β *signaling is complex and highly depends on specific protein context. In liver, we have recently showed that the disintegrin and metalloproteinase ADAM12 interacts with TGF-*β *receptors and modulates their trafficking among membranes, a crucial point in TGF-*β *signaling and development of fibrosis. The present study aims to better understand how ADAM12 impacts on TGF-*β *receptors trafficking and TGF-*β *signaling.

**Findings:**

We extracted qualitative biological observations from experimental data and defined a family of models producing a behavior compatible with the presence of ADAM12. We computationally explored the properties of this family of models which allowed us to make novel predictions. We predict that ADAM12 increases TGF-*β *receptors internalization rate between the cell surface and the endosomal membrane. It also appears that ADAM12 modifies TGF-*β *signaling shape favoring a permanent response by removing the transient component observed under physiological conditions.

**Conclusion:**

In this work, confronting differential models with qualitative biological observations, we obtained predictions giving new insights into the role of ADAM12 in TGF-*β *signaling and hepatic fibrosis process.

## Background

During chronic liver injury, the sustained wound healing response leads to fibrosis and cirrhosis. Among the molecular components involved in fibrogenesis, the transforming growth factor, TGF-*β *is the most potent profibrotic cytokine. Indeed, several authors have shown that alteration of TGF-*β *dependent signaling pathway is associated with liver fibrosis [1,2]. In this system, the first cellular control point is at the membrane. TGF-*β *transmits its signal through a heteromeric complex of two types of transmembrane receptors, T*β*RI and T*β*RII. TGF-*β *binding to T*β*RII induces recruitment and phosphorylation of T*β*RI, which activates R-Smad intracellular signaling [3]. At the membrane, an important feature of TGF-*β *signaling is the continuous internalization of receptors and ligand-receptor complexes through two endocytosis pathways: the clathrin-coated pit endocytosis, mediating R-Smad signaling cascade, and the caveolin mediated endocytosis, leading to degradation of receptors through the proteasome pathway [4,5].

We have recently demonstrated that ADAM12, a member of the disintegrin and metalloproteinase family associated with liver fibrogenesis [6,7], facilitates TGF-*β *signaling at the membrane [8]. ADAM12 interacts with T*β*RII and enhances TGF-*β *activity by modulating TGF-*β *receptor trafficking. In order to understand how ADAM12 expression might affect TGF-*β *signaling activity, we investigated mathematical models for ADAM12-dependent TGF-*β *signaling pathways. Based on models describing physiological TGF-*β *receptor trafficking and using a fitting procedure based on biological qualitative observations, we computed a family of models mimicking the pathological trafficking involving ADAM12. Their analysis allowed us to predict new effects of ADAM12 on TGF-*β *signaling.

## Results

### Mathematical models of TGF-*β *receptor trafficking

Based on two published works by Vilar *et al*. [9] and Zi *et al*. [10], we elaborated two differential models handling the trafficking of TGF-*β *receptors in membranes. We gathered the common components of Vilar *et al*. and Zi *et al*. models while putting the stress on their differences. We refer to them as V-model and Z-model for the models based upon Vilar *et al*. and Zi *et al*., respectively. The following paragraphs describe their characteristics. Table 1 and Table 2 provide a list of abbreviations and kinetic coefficients values.

**Table 1:** List of abbreviations

**abbreviation**	**definition**
*p*_*R*_	Receptors production rate
*k*_*ie*_	Internalization rate towards endosome
*K*_*re*_	Recycling rate from endosome
*k*_*ic*_	Internalization rate towards caveosome
*K*_*rc*_	Recycling rate from caveosome
*k*_*cd*_	Constitutive degradation rate
*k*_*lid*_	Ligand induced degradation rate
*k*_*a*_	LRC formation rate
LRC	Ligand-receptor complex
*R*_*m*_	Receptor complexes in the cell membrane
*R*_*e*_	Receptor complexes in the endosome
*R*_*c*_	Receptor complexes in the caveosome
*LRC*_*m*_	LRCs at the cell surface membrane
*LRC*_*e*_	LRCs in the endosome
*LRC*_*C*_	LRCs in the caveosome
CIR	Constitutive to Induced degradation Ratio

**Table 2:** Physiological values of kinetic coefficients in V-model and Z-model

	**V-model**	**Z-model**	**References**
*p*_*R *_- Receptors production rate	4	4	[9]
*k*_*ie *_- Internalization rate towards endosome	0.1667	0.1667	[5,12]
*k*_*re *_- Recycling rate from endosome	0.0333	0.0333	[12,13]
*k*_*ic *_- Internalization rate towards caveosome	0.1667	0.1667	[5,12]
*k*_*rc *_- Recycling rate from caveosome	0.0333	0.0333	[12,13]
*k*_*cd *_- Constitutive degradation rate	0.0278	0.0056	[5]
*k*_*lid *_- Ligand induced degradation rate	0.05	0.05	[5]
*k*_*a *_- LRC formation rate	1	1	[9]

#### Receptors and ligand

T*β*RI and T*β*RII are considered as a single receptor complex (named R) since the two receptors have been reported to traffic complexed together. At the cell surface membrane and upon TGF-*β *stimulation this complex can form a ligand-receptor complex (LRC). We note that homomeric T*β*RII complexes have been reported to bind TGF-*β*, however they have not shown any direct role in signaling [11] and are thus eluded.

#### Localizations

Receptors and LRCs can be present at the cell surface membrane and in internal membranes. There is a constant trafficking between those compartments which is unaffected by the presence of TGF-*β*. Internalization and recycling routes exist between the cell surface membrane and two inner compartments, the endosome *(k*_*ie*_, *k*_*re*_) and the caveosome (*k*_*ic*_, *k*_*rc*_).

#### Production and degradation of receptors

Constitutive production of receptors toward the cell surface membrane (*p*_*R*_) is balanced by constitutive degradation (*k*_*cd*_), equally affecting receptors and LRCs. LRCs present in the caveosome undergo a ligand-induced degradation *(k*_*lid*_). Constitutive degradation happens either at the cell surface (V-model) or in the endosome (Z-model).

#### Signal transmission

Signal transmission to downstream components of the TGF-*β *pathway is mediated through LRC association with R-Smads in the endosomal compartment. According to [9], TGF-*β *signaling is assumed to be proportional to the amount of LRCs internalized in the endosome.

**Figure 1:**
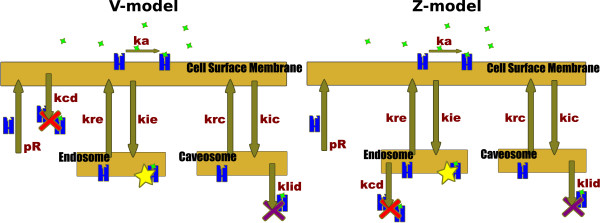
**Equations governing V-model and Z-model and diagrammatic representations**. The blue elements represent TGF-*β *receptors, the green stars are for TGF-*β*. Red and purple crosses are for constitutive degradation and ligand induced degradation, repectively. The yellow star stresses LRCs present in the endosomal compartment, standing for TGF-*β *signal strength. Abbreviations are given in table 1.

The kinetic coefficients of our two models were determined using experimental measurements [5,12,13]. Diagrammatic representations and equations are provided in Figure 1.

**Figure 2:**
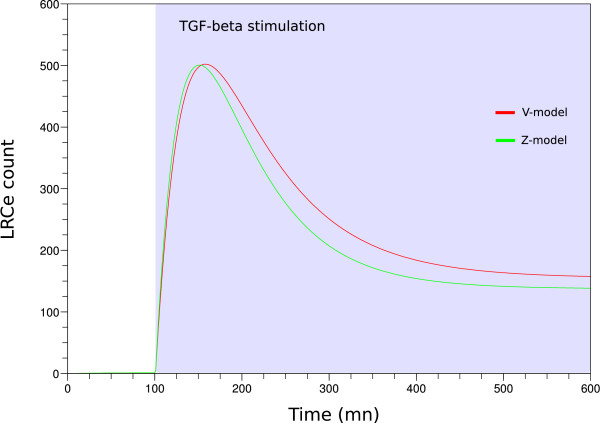
**Physiological models have similar behavior**. The evolution of the amount of LRCe present in the endosome (TGF-*β *activity) in V-model (red) and Z-model (green) have comparable trends. TGF-*β *stimulation begins at time = 100 mn.

Our first analysis was to compare the behaviors of our two models. We investigated the evolution of LRCs accumulated in the endosome upon TGF-*β *stimulation. The results are shown in Figure 2. We observe that quickly after the beginning of the stimulation, the signal raises to a high value (transient component) before decreasing to a lower stable state (permanent component). Those two models, producing very comparable behaviors, represent the physiological response of cells to TGF-*β*, in which ADAM12 is absent.

### Qualitative fitting procedure

Our biological data [8] suggest that, whether ADAM12 is present or not, receptors keep trafficking among the same compartments and are subject to the same degradation processes. We thus made the hypothesis that ADAM12 does not modify the structure of the receptor trafficking, but rather modulates the involved kinetic coefficients.

The biological data on the effect of ADAM12 are qualitative and can thus be translated into constraints that a system with ADAM12 should fulfill. We report in Table 3 the biological observations on ADAM12 used in this work, along with their mathematical translation.

**Table 3:** Biological observations and mathematical translation

	**Biological observation**	**Mathematical translation**
Constraint 1	an increase of R-Smads phosphorylation and an increase of the transcription of some SMAD regulated genes is observed upon addition of ADAM12 [8]	at stable state, with TGF-*β*, the amount of LRCs present in the endosome is increased by at least a factor 2

Constraint 2	cells transfected with T*β *RII and increasing amounts of ADAM12 proved that the more ADAM12 there was, the higher the steady state level of T*β *RII was [8]	at stable state, without TGF-*β*, the total amount of receptors present in the system is increased by at least a factor 2

Constraint 3	immunofluorescence studies showed that ADAM12 modifies the ratio of the T*β *RII present at the cell surface membrane out of those internalized, favoring the intracellular compartments [8]	at stable state, without TGF-*β*, the ratio of internalized receptors out of those present in the membrane is increased by at least a factor 2

Constraint 4	ADAM12 expression decreases the amount of SMAD7 complexed with ligand-receptor complexes, and thus their ubiquitination [8]	at stable state, with TGF-*β*, the ligand induced degradation flow is decreased by at least a factor 2

Constraint 5	overexpression studies with ADAM12 and gene reporter assay showed that ADAM12 does not maintain transcriptional activity after TGF-*β *removal (unpublished data)	after stabilization with TGF-*β*, and removal of TGF-*β*, the amount of LRCs present in the endosome becomes negligible within the first 200 minutes

**Figure 3:**
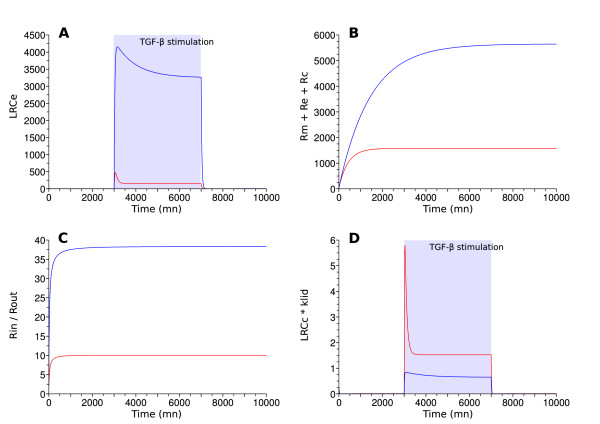
**Possible models for ADAM12 fit the constraints**. Comparison between V-model (red) and one of the models handling ADAM12 (blue). X-axis represents time in minute. The ADAM12 model has V-model architecture and its kinetic coefficient multipliers are: 7.5 *k*_*ie*_, 1 *k*_*re*_, 0.15 *k*_*ic*_, 1 *k*_*rc*_, 1 *k*_*cd*_, 1 *k*_*lid*_. Panel A displays the amount of LRCe, the system is stimulated by TGF-*β *from time = 3000 to time = 7000. The amount of LRCe, in the ADAM12 model, is increased and the signal is not persistent upon removal of TGF-*β *(constraints 1 and 5). Panel B displays the total amount of receptors present in the system, without TGF-*β *stimulation. This amount is increased in the ADAM12 model (constraint 2). Panel C displays the ratio of internalized receptors out of those in the cell membrane, without TGF-*β *stimulation. This ratio is increased in the ADAM12 model (constraint 3). Panel D displays the product of the amount of LRCe by *k*_*lid*_, the system is stimulated by TGF-*β *from time = 3000 to time = 7000. This stands for the flow of LRCe going under ligand induced degradation. This flow is decreased in the ADAM12 model (constraint 4).

We implemented a heuristic search of fitting kinetic coefficients and an exploration of the set of fitting coefficients. All kinetic coefficients except *p*_*R *_and *k*_*a *_undergo fitting. *p*_*R *_is eluded because receptor production is not significantly affected by ADAM12 and TGF-*β *stimulation (personal data). *k*_*a *_is circumvented because the formation of LRC is considered instantaneous when compared to receptor trafficking. The algorithm outputs a family of models that fits the biological data obtained in presence of ADAM12 (as exemplified in Figure 3). Models within this family only differ from V-model and Z-model by their kinetic coefficients.

### ADAM12 increases internalization from cell surface to endosome

**Figure 4:**
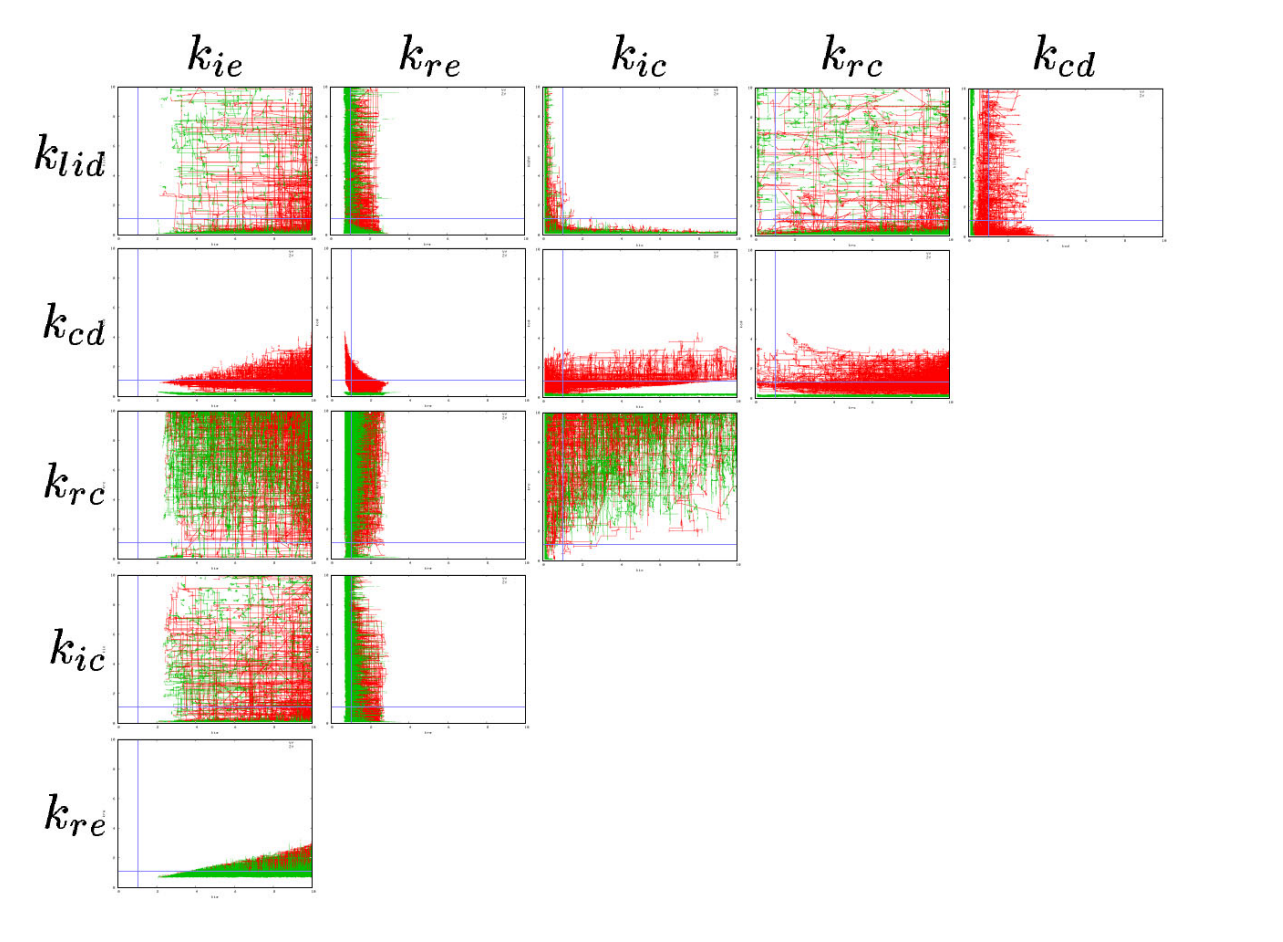
**Two-dimensional projections of the solution space**. In red are the models with V-model architecture, in green, the models with Z-model architecture. Units are the kinetic coefficient multipliers of the physiological models. Axis are scaled from 0 to 10. Blue lines represent a multiplier of 1 (physiological kinetic coefficients).

The algorithm draws kinetic coefficient lines between valid (according to experimental data) and non-valid models (Figure 4). The possible kinetic coefficients for the models fitting the effect of ADAM12 are rather similar, whether they have the architecture of V-model or Z-model.

The algorithm allows all coefficient multipliers to range from 0.1 to 10. While most coefficients present this dispersion, 3 are more tightly constrained. They are *k*_*ie*_, *k*_*re *_and *k*_*cd*_.

To take ADAM12 effect into account, the recycling rate from endosome to cell surface (*k*_*re*_) is allowed to decrease (up to 0.69 times the physiological coefficient for V-model, 0.66 for Z-model) or to increase (up to 3.08 for V-model, 3.56 for Z-model). The constitutive degradation (*k*_*cd*_) has to decrease for Z-model solutions (maximal multiplier: 0.33). The only coefficient that follows the same trend, in every solution, is the internalization towards the endosome (*k*_*ie*_), it has to increase by at least a factor 2.14 or 2.05 for V-model or Z-model architectures, respectively. Thus, the only hard prediction that can be drawn from these allowed kinetic coefficient variations, is that ADAM12 has to increase *k*_*ie *_to accord with the defined constraints. However this increase remains insufficient to explain the effect of ADAM12, as no model fits with this kinetic coefficient variation only.

Another interesting observation is that the amount of modified kinetic parameters, compared to physiological models, greatly varies from one ADAM12 model to another. Following Occam's razor, we considered the models displaying the fewer modifications. These are models following V-model architecture. They are characterized by an increased rate of receptor internalization towards the endosome and a decreased rate of internalization towards the caveosome. These modifications could be explained by a single effect of ADAM12, a redistribution of TGF-*β *receptors at the cell surface membrane, closer to clathrin pits and farther from *caveolae*.

### ADAM12 induces a permanent response to TGF-*β*

The constitutive to induced degradation ratio (CIR) characterizes the response type to TGF-*β *[9]. Upon TGF-*β *stimulation, a low CIR implies a transient response followed by an important decrease of the signal. In high CIR value systems, the decreasing phase tends to disappear and the signaling output is sustained at high values.

We computed a CIR_like _value, which generalizes the CIR properties, to characterize the reponse type of TGF-*β *under the potential effects of ADAM12. It was computed at the stable state of the models.

**Figure 5:**
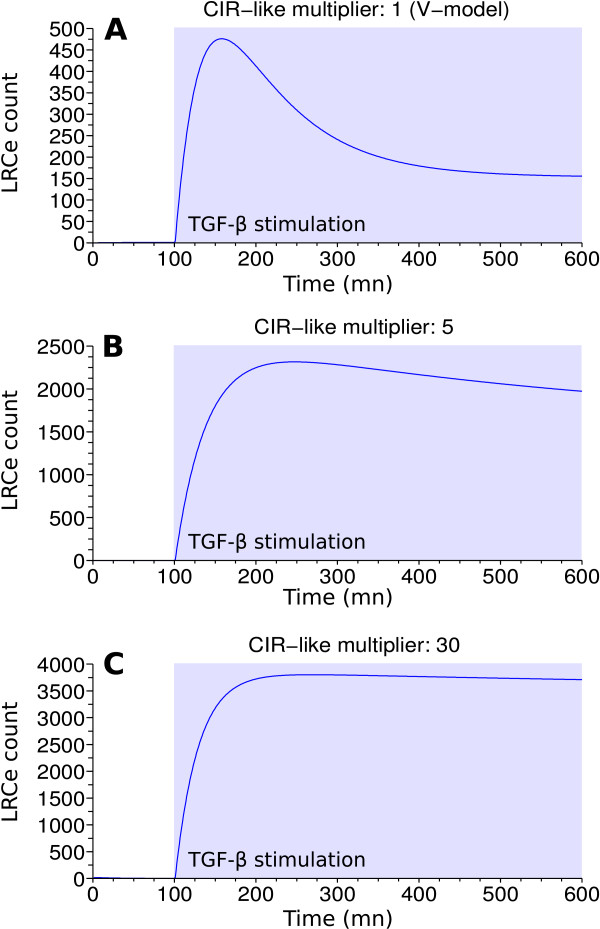
**High CIR_like _values correspond to sustained TGF-*β *signal**. The 3 panels represent TGF-*β *activity overtime. TGF-*β *stimulation starts at 100 minutes. Panel A displays the nominal V-model behavior (CIR_like _multiplier: 1). Panel B and C display ADAM12 models TGF-*β *activity, with V-model architecture. In panel B, the 5 CIR_like _multiplier is almost the minimal value that satisfies the constraints; the signal is notably more sustained than in Panel A. Panel C exemplifies a model with an higher CIR_like _multiplier (30); the signal is even more sustained.

For models with V-model architecture:



For models with Z-model architecture:



The CIR_like _value is increased in all the solutions (by a factor 3.93 and 4.43 for V-model and Z-model architectures, respectively), suggesting that ADAM12 might have an effect not only on the strength of the signal, but also on its shape. As described in Figure 5, the shape of the TGF-*β *signal is modified in pathological models when compared to the physiological ones. Panel A corresponds to V-model with its nominal parameters (medium CIR_like _value) and represents the amount of LRCs present in the endosome upon TGF-*β *stimulation, reflecting TGF-*β *signaling activity in physiological condition. Panel B and Panel C represent the response to TGF-*β *of two models handling ADAM12 (with V-model architecture). Panel B displays a system where the CIR_like _value is almost the lowest we can find among the solutions, *i.e*., 5 times the nominal value. We observe a stronger modification of TGF-*β *signaling with increasing CIR_like_, as exemplified in Panel C (for which we arbitrarily choosed a higher CIR_like _value of 30). Through these examples ADAM12 appears to induce a sustained strong activation of the TGF-*β *pathway.

## Discussion

ADAM12 is up-regulated during chronic liver injury [6,7] and modulates TGF-*β *activity [8], thereby contributing to TGF-*β *dependent liver fibrosis. In this work, we fitted differential models describing the well documented system of TGF-*β *receptor trafficking to take into account new observations on the perturbations occurring in presence of ADAM12. This led us to propose a set of possible models describing pathological situation. Studying the differences between the physiological models and the new ones, provides information about the effect of ADAM12 that could not have been drawn using only experimental observations.

Based on previous studies [9,10], we first designed differential models handling TGF-*β *receptors trafficking among membranes (V-model and Z-model). The structural difference between these models is the constitutive degradation localized at the cell surface membrane in V-model and in the endosome in Z-model. Constitutive degradation is assumed to occur at the end of the endosomal maturation process, when internalized receptors go through early endosome, late endosome and are then degraded in the lysosome. The hypothesis by Vilar *et al*. [9] implies that a sorting mechanism occurs at the cell surface membrane, separating the elements that will undergo degradation from those that will be recycled back to the cell surface. As the endosomal dynamics are still far from being fully understood, this hypothesis cannot be dismissed, and we studied the two propositions.

Using a heuristic search, driven by a cost function derived from qualitative biological data, we defined the set of possible models handling ADAM12. Those models suggest that the presence of ADAM12 increases the internalization rate of TGF-*β *receptors between the cell surface membrane and the endosome. An interesting aspect is that ADAM12 might not only affect TGF-*β *signaling strength but also its shape. This comes from the modification of the ratio between constitutive degradation and ligand induced degradation (CIR_like_), favoring the first one. In presence of ADAM12, the predicted signaling response to TGF-*β *seems like a step increase, maintaining a permanent and high level response replacing the transient component of the physiological response. Interestingly, other signaling pathways were shown to produce different responses depending on the shape of the input signal. For instance, a transient activation of the MAPK cascade in EGF pathaway leads to cell proliferation while a permanent activation leads to cell differentiation [14]. In our case, ADAM12, which according to our predictions modifies TGF-*β *signaling, could be partly responsible for the changes observed in TGF-*β *signaling during liver fibrosis.

Finally, although many models for the possible effect of ADAM12 differ from the physiological models by all the kinetic parameters, it is difficult to imagine that ADAM12 could have such a wide range of action, *i.e*. changing every parameter. In fact, some models are a lot more parsimonious. For instance, a group of them only shows an increased rate of internalization towards the endosome and a decreased rate of internalization towards the caveosome. Under such assumption, the effect of ADAM12 could be a redistribution of TGF-*β *receptors in the cell surface membrane closer to clathrine pits and farther from *caveolae*. Interestingly, Interleukin-6 (IL-6), an important regulator of inflammation, has been suggested to similarly decrease the partitioning of TGF-*β *receptors to the raft-caveolin membrane fractions while enhancing TGF-*β *signaling activity [15].

Taken together, our data give new insights into the role of ADAM12 in TGF-*β *signaling. Our models predict that its impact might contribute to the sustained TGF-*β *activity that triggers liver fibrosis.

## Competing interests

The authors declare that they have no competing interests.

## Authors' contributions

JG conceived the study, performed the computational analyses and drafted the manuscript. ML conceived the study and participated in drafting the manuscript. NL participated in drafting the manuscript. NT participated in the conception of the study, the drafting of the manuscript and brought biological expertise. All authors read and approved the manuscript.
